# A Good Work Well Finished

**Published:** 1887-01-15

**Authors:** 


### A Good Work Well Finished.

"Finis Coronat Opus."

The speech made by Mrs. Gulson at the forty-fifth annual meeting of the friends and subscribers of the Coventry and Warwickshire Hospital, when she handed over a cheque for £4,400, to be invested as an endowment fund for the permanent maintenance of a children's ward, possessed more than a local interest, and is full of suggestions and instruction to those interested in hospitals. At the suggestion of Lord Leigh, the children's branch of the Coventry and Warwickshire will be set apart, and called for ever the Gulson Wards, and the admission to the whole of the cots will be free, and without ticket. A full account of Mrs. Gulson's work will be found on page 106 of The Hospital for Nov. 13th. May many people in many places be moved to go and do likewise!

What has been done.

Mrs. Gulson said: With Lord Leigh's kind permission I wish to make a short statement regarding the Endowment Fund of the Children's Hospital, which, according to the resolve I expressed at the last annual meeting, has been happily completed. When I undertook to raise the required income of £200 a-year I soon felt that I must commence an Endowment Fund. The idea was not thought by many to have much chance of fulfilment, but, by keeping the desired end steadily in view, I secured before the late bazaar a sum of £3,827 6*s*, 9*d*., which was materially increased by the lucky investment of money from the first in my husband's hands at 5 per cent. He has now added to his great kindness to me throughout these toiling years, by giving £202 to enable me to place to-day at the disposal of your committee the sum of £4,400, as a permanent Endowment for the Children's Wards in the Coventry and Warwickshire Hospital. The only condition I make is that for which I originally stipulated, viz.: That these wards shall remain for ever free for the sick children of Coventry, and the neighbourhood, no ticket being required, when the doctor considers the case admissable. With the endowment Fund happily accomplished, there will be no further annual subscriptions required after this year. I can fancy hearing from many lips the words, "What a blessing!" I, too, can re-echo the sentiment, and from my heart I feel thankful that, to myself as well as to those true and loyal helpers who have so nobly worked for and with me in this good cause, time has brought to us the end of our labours.

Room for Help Still.

Still there is room for help in the future, besides the annual income I have always provided for the various wants of the little ones and a Samaritan Fund will still be required to send our sick children to convalescent homes, for blankets and sheets, and for those little red jackets you all know so well. Therefore, I hope whenever anyone has a little money to spare they will think of our sick children. I would also hope that the annual collections in the Sunday Schools may be continued. The interest already awakened on behalf of the little sufferers should not be extinguished in the hearts of their more fortunate brothers and sisters, and will be to them twice blessed—"It blesseth him who gives and him who takes."

Thanks to Others.

I wish now to express my heartfelt thanks to my valued friends and helpers for so many years, Miss Grimes and Miss Sargeant, to whose unwearied energy and kindness I am indebted for the assistance they have given me in securing the annual income, at a cost of great personal exertion and fatigue. I have also to thank Mrs. Graham, Miss Kirby, Mrs. Nutt, Mr. Linnell, Mrs. Hands, of Canley, and Mrs. C. Waters,—who have all assisted me at various times in the same arduous work. I have also to express my most grateful acknowledgments to the Coventry Press for the liberality with which they seconded all my efforts by every means in their power, and by inserting all my advertisements at half-price.

The Bazaar.

The bazaar receipts were £472 15*s*. 8*d*.; donations, £143 3*s*.; making a total of £615 18*s*. 8*d*., from which the expenses are probably about £250, leaving a balance of £369 18*s*. 8d. I have had the kindest help and sympathy from all classes in this great city, and to all I offer my sincere and grateful thanks. It has been impossible to do so individually. I owe a deep debt of gratitude to Lady Brooke, for coming, at considerable inconvenience, to open the bazaar with so much kindliness and grace; and to Mr. Eaton, M.P., and to his son Colonel Eaton, I am materially indebted for the great success of the bazaar; and to Mrs. and Miss Dewes, Mrs. Waters and her daughters, Mrs. C. Hill and Miss Hill: and to Mr. Charles Browett and Mr. G. Stewart, I cannot sufficiently express my obligations. To all the ladies and gentlemen who so actively assisted me, both at the stalls and in the entertainments held during the week, I wish to convey my deep sense of the value of their most untiring and admirable zeal.

Well done All.

To the Mayor of Coventry and to his predecessors in office, I have much reason to be grateful, as they have always shown me the kindest consideration on every occasion when their patronage was needed. The Committee will, I hope, believe how much I thank them for their courtesy in acceding to every reasonable application I have made to them; and in now placing the wards in their hands I feel assured of their loving and watchful guardianship over the trust committed to them. I cannot complete my retrospect without referring to the invaluable services of Nurse Walker, whose rare devotion to the children she tended is well-known to many here to-day. She has been followed by our present excellent Nurse Gilbert, to whose patient care of the little ones I know all our doctors will bear a willing testimony. Illness has removed from Coventry one of the children's best and kindest friends, Miss Muriel, whose weekly visits were looked forward to with the deepest interest. Will not others try to fill her place? We want visitors to brighten up a short period of many weary days to our little patients. The cause of the sick children of Coventry will always be dear to me, and I can never cease to care for their welfare and to superintend their best interests. There is always a shadow of sadness in the word "Farewell," but in this case I do not feel there is anything but the shadow, through which I can look forward to the never-ceasing reward of the sick child restored to health and happiness, and to the maintenance of those kindly relations with my old friends and subscribers of all ranks which have for thirteen years cheered and encouraged my efforts towards the desired haven which I enter to-day.

